# Key aspects defining the development and implementation of a regional genomic surveillance strategy for the Eastern Mediterranean Region

**DOI:** 10.1111/irv.13205

**Published:** 2023-10-18

**Authors:** Luke W. Meredith, Mustafa Aboualy, Rachel Ochola, Patrick Okwarah, Mehmet Ozel, Abdinasir Abubakar, Amal Barakat

**Affiliations:** ^1^ Infectious Hazard Management, Department of Health Emergency World Health Organization, Eastern Mediterranean Regional Office Cairo Egypt

**Keywords:** bioinformatics, genomics, influenza, next‐generation sequencing, SARS‐CoV‐2, whole genome sequencing

## Abstract

The COVID‐19 pandemic highlighted the critical role of pathogen sequencing in making informed public health decisions. Initially, the Eastern Mediterranean Region faced limitations in sequencing capacity. However, with robust WHO and stakeholder support, the situation significantly improved. By 2022, COVID‐19 sequencing was underway in 22 out of 23 regional countries, with varying throughput and capacity. Notably, three genomic hubs were established in Oman, UAE, and Morocco, playing a key role in providing expanded genomics training and support across the region. While primarily for COVID‐19 surveillance, this sequencing capacity offers an opportunity to integrate genomic surveillance into existing networks. This integration can enable early detection and response to high‐threat pathogens with pandemic potential. To advance this, WHO/EMRO collaborated with stakeholders to formulate the Eastern Mediterranean Regional Genomic Surveillance Strategy for Emerging Pathogens of Pandemic Concern. Consultative meetings with regional and international genomic surveillance experts identified strategy focal points, key partners, priority pathogens, and implementation steps. As the strategy awaits member states' ratification in Q4 2023, this manuscript outlines pivotal facets defined by member states and the strategic document's key deliverables and opportunities. These efforts aim to yield a substantial positive impact within the region.

## 1 INTRODUCTION

The growing understanding of genomic sequencing information and the rapid availability of viral sequences makes genomic sequencing a vital tool for disease surveillance.^1^ Timely and in‐depth pathogen characterization supports public health interventions, allowing for targeted and effective control of disease outbreaks.^2^, ^3^, ^4^, ^5^, ^6^ Genomic sequences can also help with the design and quality control of diagnostic assays, drugs, and vaccines by monitoring changes in the virus genome caused by evolutionary dynamics that may affect efficacy.^7^, ^8^, ^9^, ^10^, ^11^, ^12^

Whole genome sequencing (WGS) of severe acute respiratory syndrome coronavirus 2 (SARS‐CoV‐2) has been a powerful tool for monitoring SARS‐CoV‐2 since the first sequence was published on January 10, 2020.^13^, ^14^ The analysis of SARS‐CoV‐2 using robust and increasingly affordable next‐generation sequencing (NGS) technologies^15^ has been used to complement, augment, and support strategies to reduce the burden of COVID‐19.^16^, ^17^ It continues to inform improved public health policies through monitoring, detecting, and characterizing SARS‐CoV‐2 variants.

In May 2021, the World Health Assembly (WHA) urged countries to increase their capacity to detect new threats beyond COVID‐19, including through laboratory techniques such as genomic sequencing.^18^ Recognizing the global momentum and drive for investment and continual improvements in the cost, ease, and speed of sequencing,^19^ WHO released the 10‐year global genomic surveillance strategy for pathogens with pandemic and epidemic potential to support the expansion and integration of genomics into national, regional, and international pathogen surveillance programs.^20^ The implementation of this strategy aims to improve the speed of detection of pathogens as they emerge by encouraging collaboration between member states (MS) for avenues such as sample and data sharing, improving logistics and training programs and improving the integration of genomic data into existing pathogen surveillance networks.

Developing this global roadmap will require national and international cooperation, with a focus on developing countries and the global South. The UNESCO World Academy of Science advisory committee highlighted that 55% of the genomes in the Global Initiative on Sharing All Influenza Data (GISAID) platform come from the United Kingdom or United States, which account for 5% of the global population.^21^ A review of the GISAID repository shows that Africa, Asia, the Middle East, and South America combined have contributed 5% of the genomes, illustrating the disparity.

Regional networks, centers for excellence, and multi‐sectoral collaboration between national public health institutes, academia, and commercial laboratories will be required to build sustainable genomic capacity, sharing costs, data, expertise, and infrastructure to support regional public health. In this paper, we document the approach and steps taken by the WHO Regional Office of the Eastern Mediterranean (WHO/EMRO) and MS of the Eastern Mediterranean Region (EMR) to develop and operationalize the Regional genomic surveillance strategy in the EMR, in‐line with the Global genomic surveillance strategy.

## 2 FOUNDATIONS

### 2.1 Landscape review of regional genomic sequencing capacity during the COVID‐19 pandemic

The WHO/EMR comprises the occupied Palestinian territory and 21 MS: Afghanistan, Bahrain, Djibouti, Egypt, Iran, Iraq, Jordan, Kuwait, Lebanon, Libya, Morocco, Oman, Pakistan, Qatar, Saudi Arabia, Somalia, Sudan, Syrian Arab Republic, Tunisia, United Arab Emirates, and Yemen. They have diverse cultures, socio‐economic conditions, and demographic characteristics. The provision of health and other services in the region is challenging due to acute and protracted humanitarian emergencies, poverty, political instability, and fragile health systems.^22^, ^23^, ^24^

An assessment of MS capacity has been conducted over the past 24 months, both before and during the COVID‐19 pandemic. The results showed a wide range of capacity and investment, with diverse regional capacities. This is not unexpected, as countries have a wide range of socioeconomic conditions and health and emergency responses that place stress on existing infrastructure, affecting the ability to respond to new crises as they emerge. By the time the pandemic was declared in 2020, 11 countries had invested proactively in capacity for routine genomic surveillance through National Public Health Laboratories and National Influenza Centers (NIC). An additional four countries provisioned genomic capacity for COVID‐19 in 2021, investing in sequencing equipment, training, data, and sample management.

Three regional reference laboratories were established during the COVID‐19 pandemic, with strong logistical and technical links to countries across the region, to provide support for genomics and sample testing, continued training and support with bioinformatics and data analysis. These are Sheikh Khalifa Medical City (SKMC) Reference Laboratory for Infectious Diseases, United Arab Emirates; Laboratoire de Virologie at the Institut National d'Hygiene in Morocco; and the Central Public Health Laboratory in Oman. These laboratories will be supported both nationally and by the WHO/EMRO to continue expanding capacity for sequencing, with the goal of having recognized reference laboratories for high‐risk pathogens that can facilitate training and testing support for countries as they develop their in‐country capacity.

Of the remaining seven countries, while sequencing capacity was a priority, conditions were not conducive to a rapid investment or expansion of sequencing laboratories due to complex emergencies, conflicts, and logistical challenges associated with getting reagents or samples into or out of the country. Lack of cold‐chain and courier capacity for sample transport, limited information technology infrastructure, trade restrictions, and a lack of expertise in sequencing, both from the laboratory protocols, bioinformatics and interpretation of genomic data, resulted in delays in the implementation of sequencing in these countries, presenting a clear health risk to the populations (Table 1).

**TABLE 1:** The key challenges and opportunities defined by the collaborative meetings in the Eastern Mediterranean Region.

Challenges	Opportunities
**EMR laboratory genomic‐specific challenges**	
Weak supply chain capacity	Best practices for forecasting and procurement need to be developed and adapted
Weak sample packaging and transportation capacity	Investments to improve sample transportation domestically and internationally to reduce turnaround time, respect cold chain, and increase testing
No point of care (POC) tests	POC technologies key for decentralizing tests and expanding access
Laboratory equipment (availability and maintenance)	Explore the possibility of procuring only from companies based within the country or the region to ensure availability of long‐term maintenance
Quality assurance	Re‐emphasize the importance of participating in an external quality assessment program (EQAP) and continue providing support for the provision of EQAP panels
Inadequate human workforce for laboratories	Support short‐ and long‐term trainings to build local capacities on basic and advanced laboratory diagnostics
Lack of domestic capacity to manufacture diagnostic tests in the region	Consider pooled procurement and distribution to countries from WHO Dubai Logistics Hub
**EMR operational challenges**	
Limited availability of lab equipment/kits in the local market	Stockpile of kits for emerging epidemic and pandemic‐prone infectious diseases in the Dubai Logistics Hub
Slow procurement mechanisms	Consider expansion of WHO catalog to include wide range of emerging epidemic and pandemic‐prone infectious diseases kits to hasten procurement mechanisms currently hampered by the lack of these items; short shelf‐life of emerging epidemic and pandemic‐prone infectious diseases kits
Limited funding	The consideration by national disease control programs, emergency responses and other surveillance programs of genomic surveillance as an integral part of the broader public health and thus commit sufficient resources to it.
Embargo for some countries	Considering limited cold chain storage at country level

**FIGURE 1:**
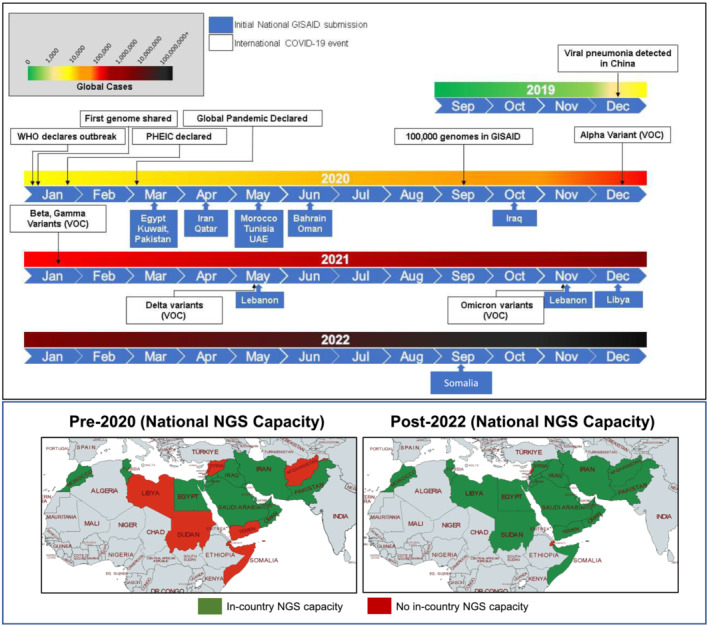
NGS capacity for SARS‐CoV‐2 surveillance has evolved rapidly in EMR and is now supported in 21/22 countries in the region. WHO/EMRO, with the support of national and international stakeholders supported the expansion of NGS capacity for SARS‐CoV‐2 sequencing across the EMR. Within 6 months of the pandemic declaration, 10 countries were actively contributing to GISAID, and by the end of 2022, 21/22 countries had deposited data into the repository.

WHO/EMRO, in collaboration with international stakeholders, prioritized support for these resource‐limited countries (Afghanistan, Iraq, Lebanon, Libya, Somalia, Sudan, Syria, and Yemen) to establish sequencing capacity in National Public Health Laboratories using Oxford Nanopore Technology.^25^, ^26^, ^27^, ^28^ This portable NGS platform is easy to use in any environment, enabling real‐time DNA and RNA sequencing and analysis inside and outside the laboratory. In‐country and regional training in both laboratory techniques and bioinformatics were provided by international experts and stakeholders, including the UKHSA and US CDC. As a result of this investment in capacity, seven of these countries were actively sequencing by the end of 2022 and have started contributing or are intending to contribute to global repositories in the next 3 months. An exception is Sudan, who are currently experiencing severe civil unrest and consequently have limited access to laboratory infrastructure. An overview of all countries with national sequencing capacity pre‐ and post‐2020 is shown in Figure 1.

**FIGURE 2:**
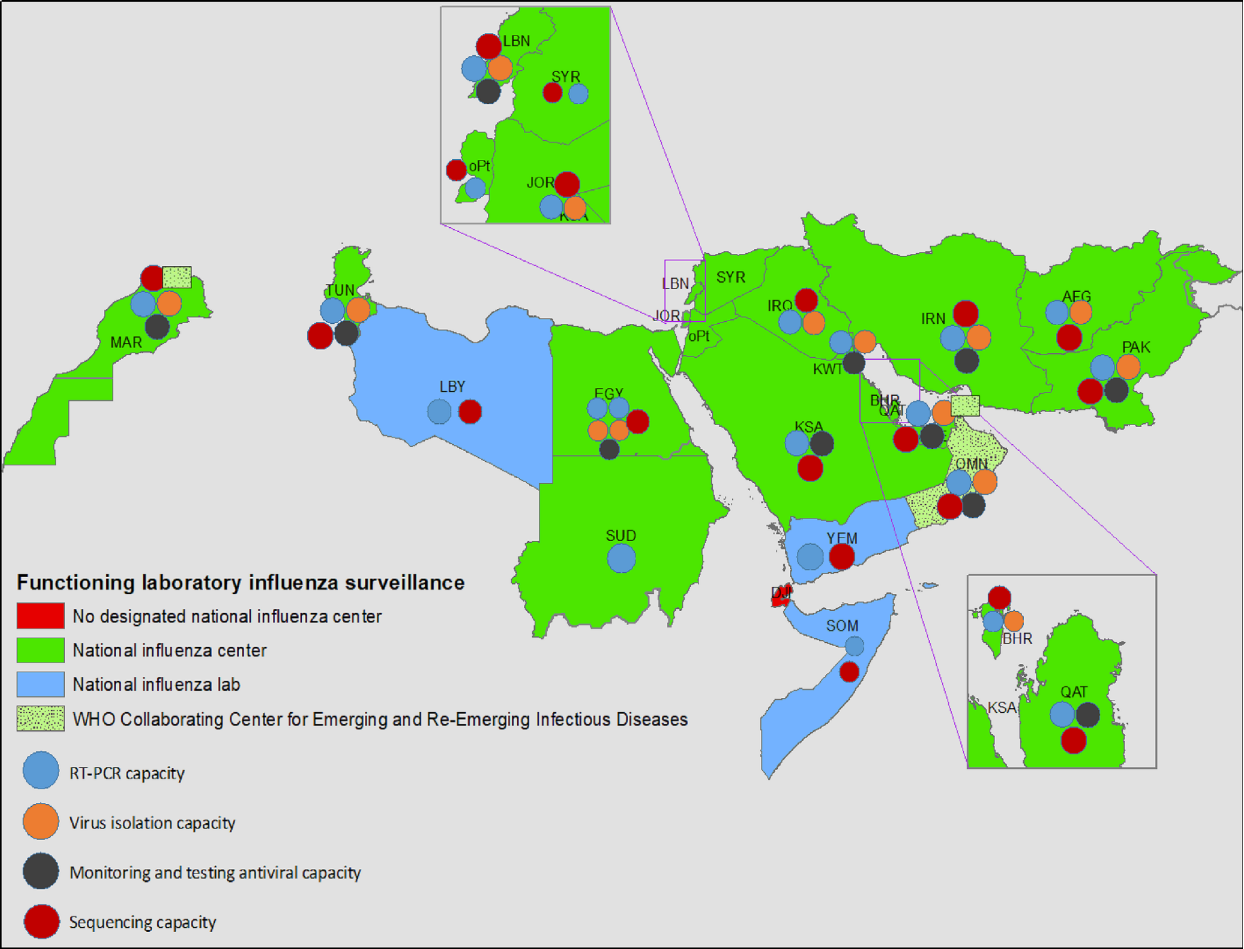
An overview of reference laboratories and WHO collaborating centers across the Eastern Mediterranean Region. Reference laboratories, national influenza centers, and WHO collaborating centers have been set up across the region to actively support genomic surveillance. The map indicates facilities with NGS, PCR, and virus isolation capacity.

A timeline for when countries began sharing data internationally through the GISAID database indicates regional contributions and preparedness (Figure 1). WHO declared COVID‐19 a pandemic in March 2020, and within 3 months, national and sub‐national laboratories from 10 countries were depositing genomes to GISAID. While this serves as a response metric, other countries worked with international collaborators in Europe, United States, and Asia, ensuring the region contributed to the global response (Figure 2). By the end of 2022, 15 countries were depositing sequences to GISAID, emphasizing the improvements in national capacities for COVID‐19.

### 2.2 Consultative fact‐finding to determine member state opinions and requests of a genomic surveillance network

The key aim of the regional genomics strategy is to take advantage of the investment in genomics capacity rolled out during the COVID‐19 pandemic to establish a sustainable regional genomic surveillance network for high‐threat pathogens. This network would directly respond to many of the challenges found in Table 1 and allow for better coordination and information sharing for quality, timely, and appropriate public health actions locally and globally.^29^, ^30^ For this to be successful and to encourage membership to the strategy, it is important that MS goals and feedback need to be taken into consideration when developing the network. As mentioned previously, the diverse range of socio‐economic conditions means that national capacity varies greatly across the region and support needs to be given at all levels to ensure tangible outputs are achieved by the strategy.

While a framework of the regional genomic surveillance strategy was in development, a regional meeting took place in Amman, Jordan, between 1 and 2 February 2023, under a title of “Genomics technical workshop to explore a strategy and roadmap for the creation of a sequencing surveillance network in EMR for emerging and re‐emerging infectious diseases.” WHO/EMRO, regional and international partners, including directors and managers of human, veterinary, and environmental health agencies from MS attended the meeting to review developments on the strategic framework, provide feedback on goals, objectives, and key activities, including operational steps that would encourage regional participation and implementation of the strategy. Other key international stakeholders include (but are not limited to) funding agencies, such as the World Bank, Pandemic Fund, donors such as the Rockefeller Foundation, international agencies such as World Organization for Animal Health (WOAH), Food and Agriculture Organization (FAO), US Center for Disease Control and Prevention (US CDC), and UK Health Security Agency (UKHSA). Cross‐sector collaboration was encouraged by the inclusion of academic institutions and commercial laboratories.

The full report on this meeting will be released through the WHO website in Q4, but the key findings and challenges are summarized in (Table 1). Network‐level support to drive engagement and national policy advocacy supporting the network, data sharing, harmonization of training and operating protocols, and increasing group bargaining power for purchasing and logistics are all key aspects requiring support, and tangible recommendations supporting these will need to be incorporated into the strategy.

**FIGURE 3:**
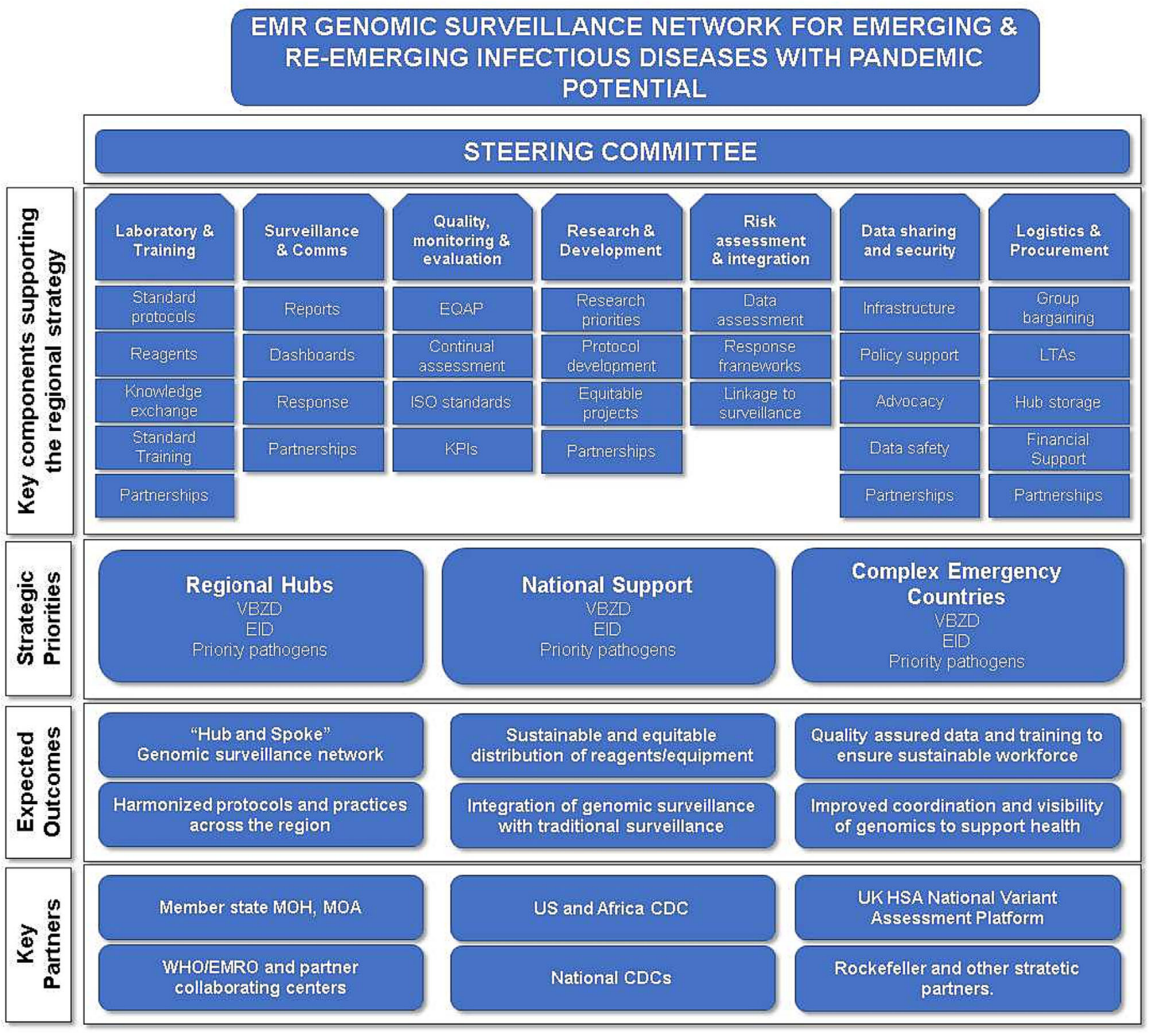
Proposed operational structure for the EMR Genomic Surveillance Network. The regional genomic strategy aims to support a multi‐sectoral, cross‐cutting genomics surveillance network, with collaborative input from MS and stakeholders. A steering committee providing high‐level strategic and policy guidance, composed of multi‐sectoral experts in genomics, health policy and strategy, and pathogen surveillance, will be recommended. Pillars to support the technical operation of the network will be convened, with MS and stakeholder experts in each pillar to ensure achievable key performance indicators are established and routinely achieved. Maintenance and expansion of regional and national hubs will be strategic priorities, as will continued expansion of support for complex emergency countries. Outcomes and partners will be defined and expanded on in the strategy, for ratification with MS once a draft strategy is finalized.

MS requested that the strategy advocates for establishing steering committees and technical working groups to help with the definition and operationalization of both the strategic network and national capacities. Several pillars were proposed, with each needing defined goals and key performance indicators, as outlined in Figure 3, contributing to the overarching goal of ensuring that a sustainable regional genomic surveillance network for emerging or re‐emerging pathogens that can respond rapidly to outbreaks as they occur and to ensure genomics is integrated into the public health framework to provide support for decision making in the region and globally. Stakeholders will provide input and support to specific goals, all working to ensure the network is sustainable. Priority support areas, including building national capacity, supporting regional hubs, and specific support for countries with complex emergencies, are considered key.

MS also observed that the outcomes of the strategy and network needed to be clear and tangible to encourage membership. Continued expansion of existing country capacities, such as trained workforces operating with GISRS and NICs, and the expansion of capacity for non‐respiratory pathogens designated as priorities within the region. A clear priority of the MS was focusing on an integrated, cross‐cutting approach to surveillance, incorporating food safety, agriculture, environmental genomics, and public health, with a true One Health approach to the strategy.

In response to these observations, a list of priority pathogens was assembled by the attendees through group discussion and consensus, covering high‐threat pathogens of regional concern. While the formal list needs to be ratified before publication, the breadth of responses highlights that there are a broad range of concerns in the region, ranging from bacterial (causing acute watery diarrhea, or food safety concerns), viral (including respiratory pathogens, but also arboviral such as dengue fever, or hemorrhagic fever causing viruses such as Crimean Congo Hemorrhagic Fever that threaten both human and animal health) and parasitic pathogens (including malaria and schistosomiasis) that threaten human, agricultural, and animal populations. The optimal pathways to ensure surveillance of this range of pathogens needs to be considered, as at present, there is no single laboratory in the region with the capacity to sequence all the pathogens listed, making this a key focus of the developing strategy.

## 3 APPROACHES AND KEY DELIVERABLES FOR DEVELOPING THE REGIONAL GENOMIC SURVEILLANCE STRATEGY

### 3.1 Regional genomics surveillance strategies should align with both international and national strategies and priorities to strengthen, rather than dilute, collaboration

Based on the landscape review, consultative meetings, and regional experience, a series of key principles for development and subsequent implementation of a regional genomic surveillance strategy were assembled. A key goal of the network will be the capacity to rapidly respond to high‐threat, priority pathogens as they emerge or re‐emerge in the region. Attempts have been made previously to establish these networks, with varying degrees of success, and lessons need to be learned from the successes or limitations of these networks, and the feedback gathered in the consultative meetings has highlighted the key issues being faced by all genomics projects in the region. Sample management and logistics, reagent logistics, establishment of minimal data and metadata requirements, data collection and analysis training, harmonization of training materials, and operating procedures are recurring themes in MS member state feedback, and broadly aligns with the recommendations outlined in the Global Genomic Surveillance Strategy for Pathogens with Pandemic and Epidemic Potential 2022–2032,^20^ (referred to as the Global Genomic Strategy for brevity from here on).

The Global Genomic Strategy was established based on input from experts in the human, animal, and environmental health sector and strongly advocates for cross‐sectoral engagement between governments, intergovernmental agencies, global, regional and national networks, technical partners, donors, academia, and the private sector. It provides high level guidance on the operation of a sequencing network, with guiding principles that need to be adapted to reflect the situation in regional and national settings. This strategy has been considered during the operationalization of several initiatives, including the International Pathogen Surveillance Network (IPSN),^31^ BioHub,^32^ and other pathogen‐specific initiatives, and the regional strategy will seek to align as closely as possible with the recommendations within this guidance to streamline interactions with international partners both within and external to WHO.

While the developments of both the regional and international genomics strategies are important milestones in the expansion of genomic surveillance, it is important to maintain focus on the priority of ensuring the strengthening of national surveillance systems, as the first line of response to pathogens as they emerge. This is particularly important with changes in funding policies from USAID and the implementation of the Pandemic Fund, which provides funding avenues for projects managed at the national level, rather than regional or international. With this focus in mind, WHO/EMRO is collaborating with both WHO International and MS to deliver training and support for the development of national genomic surveillance strategies containing clearly defined goals and deliverables, supported by detailed costing analyses to ensure the long‐term sustainability of genomics in MS, while also aligned closely with both the regional and international strategies.

### 3.2 Genomic surveillance needs to be cross‐cutting and inclusive, with a One Health approach to monitor the emergence of high‐threat pathogens

A key lesson from the COVID‐19 pandemic is that pathogens can emerge from nature more at any time. The sequencing strategy needs to incorporate a “One Health” approach to improve linkage between sectors, including human, veterinary, food health, and environmental sectors, ensuring that genomic information draws on a wide network of sampling strategies and improves surveillance outcome. This will also improve the sustainability of genomics in the region by expanding the available pool of talent and expertise for sequencing.

With this objective in mind, several key action points need to be finalized before the regional strategy can be implemented. Initially, the pathogens of concern need to be established and ratified by the MS. The inclusion of specific pathogens in the strategy has a threefold purpose: First, this will allow for a secondary landscape review of pathogen‐specific surveillance networks, to ensure that the sequencing and genomic data provide a useful benefit to public health responses, and second to guide the implementation of protocols, training packages, and bioinformatics analyses that are needed to operationalize sequencing at the national or regional level. And third, this will aim to minimize the duplication of effort between technical working groups and other already established networks, to meet the observations of the previous section regarding collaboration and cooperation between teams.

To ensure that the genomic surveillance network incorporates as many partners as possible, and in accordance with the results of the consultative meetings, a key deliverable of the strategy will be the convening of a steering committee for regional genomic surveillance. This committee should be comprised of high‐level experts in policy for areas including public health, agriculture, environment, and legal experts with knowledge of complex areas such as cross‐border data sharing, which are crucial to the function of a genomic surveillance system. Members of this committee should be drawn from the region, to ensure knowledge of local conditions, but be supported by international experts with experience and expertise in disease surveillance, including genomic surveillance. The purpose of the committee will be to provide high‐level guidance to MS and the network on goals, funding, key performance indicators, and the direction the network should take to implement policy across the region. It should also provide advocacy at the highest possible levels to ensure that MS are engaged with genomic surveillance, to ensure the sustainability of genomics in the region.

The steering committee should be supported by technical working groups, composed of national and international subject matter experts in areas of critical importance to the function of genomic surveillance (outlined in Figure 3). While some of the areas are clear, including technical areas such as protocol development and implementation, documentation, quality assurance, training, and harmonization, other areas require significantly more development, particularly data sharing agreements and the implementation of Memoranda of Understanding to support sharing and transfer of data, expertise and equipment throughout the network. The working groups and steering committee should work together to drive genomics throughout the region and to maintain the focus on the vision of integrating a cross‐cutting, One Health approach to genomic surveillance with existing surveillance networks.

### 3.3 The network should capitalize and expand on existing development and investment in sequencing throughout the region by implementing a “hub‐and‐spoke” style genomic network

As evidenced by the landscape analysis, there is a significant amount of sequencing capacity now available in the EM region. While the strategy aims to expand the scope of genomics in the region, it will look to build on the existing capacity established both before and during the COVID‐19 pandemic.

A case in point for this expanded capacity was the implementation and operationalization of the three regional genomics hubs, established in United Arab Emirates, Oman, and Morocco. These facilities have a high level of technical expertise, capacity, and knowledge, which has been applied to support the region throughout the pandemic. As the burden of COVID‐19 has eased in previous months, the focus is now turning to how to further expand the capacity of these laboratories to support the region for surveillance of other pathogens.

While most countries now have capacity for sequencing, it is not feasible in the short term to roll out training and support for 10 or more pathogens to all national systems simultaneously. The strategy instead aims to operate on a “hub‐and‐spoke” model, with central locations acting as reference and support laboratories (“Hubs”) providing support, training and monitoring to decentralized, national locations (“Spokes”) who over time will develop standalone capacity and expertise.

As mentioned in the previous section, there is an extensive list of pathogens of concern for the region. The strategy aims to capitalize on the regional hubs by allocating specific pathogens to each hub based on the needs of the region. While the steering committee will have the task of finalizing the allocations, it is envisioned, for example, that a hub could be responsible for viral hemorrhagic fevers sequencing. The hub would then be the primary focus for training with international partners, such as the US CDC or UKHSA NVAP program, providing intensive support for areas operationalization of laboratory and bioinformatic protocols, surveillance and sampling strategies, risk assessments, and reporting frameworks to ensure that each hub can function as a reference laboratory for the pathogen of concern.

While functioning primarily as referral laboratories, the hubs would also be responsible for supporting the region in a wider scope, bringing the ownership of the processes and protocols directly into the region, rather than relying on support from external agencies, who are often under extreme pressure due to the wide range of responses they undertake. This would also improve the cost efficiency and sustainability of sequencing, as training courses would be managed directly by the region and countries would be operating with consistent protocols and reagents allowing for improved bargaining capacity when approaching suppliers. Regional quality would also be improved, as auditing expertise would be in the hubs, allowing for more frequent, sustained assessment of quality in the region.

### 3.4 Focusing on quality, monitoring, and evaluation of key performance indicators will not only ensure high‐quality data but build trust in genomic surveillance as a tool for public health

A surveillance system with a well‐defined sampling and sequencing strategy, including integrated genome sequencing, will ensure the systematic nature of sample collection, representativeness, quality of genetic sequencing data,^33^ completeness, timeliness, reliability of data generated, continuity, and sustainability of the sequencing activity as part of ongoing national surveillance. Quality control will be advocated for at multiple levels, including at the laboratory level, by following recommended good laboratory practices, enrollment in EQAP, providing regular assessment of staff training and competency, and providing support for continuing professional development of staff to ensure a sustainable workforce in the longer term.

Beyond the laboratory, bioinformatic quality assurance will also be supported and monitored. The implementation of standard quality checks on raw data and using protocols shared across the region will ensure trust in the data and genomics as a tool. Support for bioinformatics training and continued knowledge sharing and exchange across the region will be encouraged and facilitated by regional and international stakeholders to build a cohort of bioinformatics expertise for the network.

Bioinformatics expertise has been identified as a critical aspect requiring support, and continued investment in training, standardization, and infrastructure will benefit the region in the long term. Training for regional hubs, training‐the‐trainer initiatives, staff exchanges, and twinning initiatives with collaborative centers for pathogen surveillance will continue to build on existing expertise beyond COVID‐19. Training will be multi‐sectoral ensuring that key stakeholders in human and veterinary health, as well as surveillance and policy experts, know how genomic data are generated and can be used for supporting health responses.

Key performance indicators will be established, with metrics agreed upon and ratified by MS involved in the network. Suggested metrics include sample processing and turnaround time, integration with surveillance networks, risk assessment, and alert responses, EQAP results, staff competency, and retention, with an overriding metric of providing a rapid response to pathogens as they emerge.

## 4 DEVELOPMENT, PRESENTATION, AND IMPLEMENTATION

Over the past 12 months, the key elements outlined in this manuscript have been incorporated into a document entitled “Eastern Mediterranean Regional Genomic Surveillance Strategy for Pathogens with Pandemic and Epidemic Potential.” This strategic document outlines the vision for the surveillance network and outlines the key elements that will be critical to the successful implementation of genomic surveillance in the region. This document has been submitted for internal review, with the goal of presenting the strategy to the region for further consultation and consideration at a meeting to be held in October of 2023. At this meeting, select MS will be given another opportunity to review and ratify the strategy for the region.

Once the strategy is ratified, it will be published for distribution, while the implementation plan will be developed based on the strategy. The key aspect of the composition of the steering committee and technical work groups will be discussed, with proposals for membership presented by stakeholders from the region.

## 5 CONCLUSION

At the onset of the COVID‐19 pandemic, GISRS network was leveraged to integrate SARS‐CoV‐2 into sentinel systems for influenza‐like illness, acute respiratory infection, and severe acute respiratory infection to inform policy and the national responses to the outbreak for sustainable and reliable analysis and interpretation of genome data. WHO/EMRO has made substantial investments in NGS and the computing infrastructure required for incorporating pathogen genomics into public health in EMR. The aim of the EMR genomic surveillance strategy is to build on the gains made by countries thus far and in parallel, equip the remaining laboratories in complex countries with much‐needed capacities and capabilities for genomics sequencing.

WHO/EMRO continues to provide leadership, oversight, and coordination to enhance sustainable surveillance systems for emerging epidemic and pandemic‐prone infectious diseases. In doing so, countries will have an established framework for effective genomic surveillance strategy and capacity‐strengthening plans allowing for a clear set of priority genomic surveillance use cases. Use cases could include avenues such as defining sampling strategies, protocols, analyses, and reporting strategies to guide implementation of genomics in a timely manner. Training centers and workforce training programs for sequencing and bioinformatics, quality assurance programs for sequencing and bioinformatics, sequencing ‘nodes’ at the country level (where feasible) and regional referral hubs will establish communities of practice. This will also lead to better access to supplies and consumables, including data management solutions.

EMR has gained immense experience and insight as innovations have impacted program performance over time, and the lessons learned should be considered a valuable resource. Therefore, the performance of an ever‐evolving public health system emphasizes the need for continual improvement, connectivity, and close monitoring.^34^ In conclusion, challenges notwithstanding, the feasibility of realizing an expanded near real‐time regional genomics surveillance system for multiple pathogens will better prepare EMR countries for the next big outbreak without any significant negative impact on existing surveillance systems.

## AUTHOR CONTRIBUTIONS

Abdinasir Abubakar, Amal Barakat, Rachel Ochola, and Mehmet Ozel conceptualized the paper. Luke W. Meredith drafted the manuscript with assistance from Mustafa Aboualy, Rachel Ochola, and Mehmet Ozel. Patrick Okwarah provided editing and proofing support. All authors reviewed and contributed to subsequent drafts for important intellectual content and approved the final manuscript. The authors alone are responsible for the views expressed in this publication, and they do not necessarily represent the decisions or policies of WHO/EMRO.

## CONFLICT OF INTEREST STATEMENT

The authors declare that they have no financial or personal relationships which may have inappropriately influenced them in the writing of this article. All authors are employed by WHO/EMRO.

## Data Availability

Data sharing is not applicable to this article, as no new data were created or analyzed in this study.
